# Supplementary material for: Pathogenic KIF1A variants differentially disrupt axonal trafficking and impede synaptic development
Source: bioRxiv. 2026 Jan 14:2026.01.14.699478. Preprint. [Version 1] doi: 10.64898/2026.01.14.699478 (PMC12871302; doi:10.64898/2026.01.14.699478)
Supplement: 1 [file NIHPP2026.01.14.699478V1-supplement-1.pdf]

bioRxiv preprint doi: https://doi.org/10.64898/2026.01.14.699478; this version posted January 14, 2026. The copyright holder for this preprint (which was not certified by peer review) is the author/funder, who has granted bioRxiv a license to display the preprint in perpetuity. It is made available under aCC-BY 4.0 International license.

Supplemental Figure 1

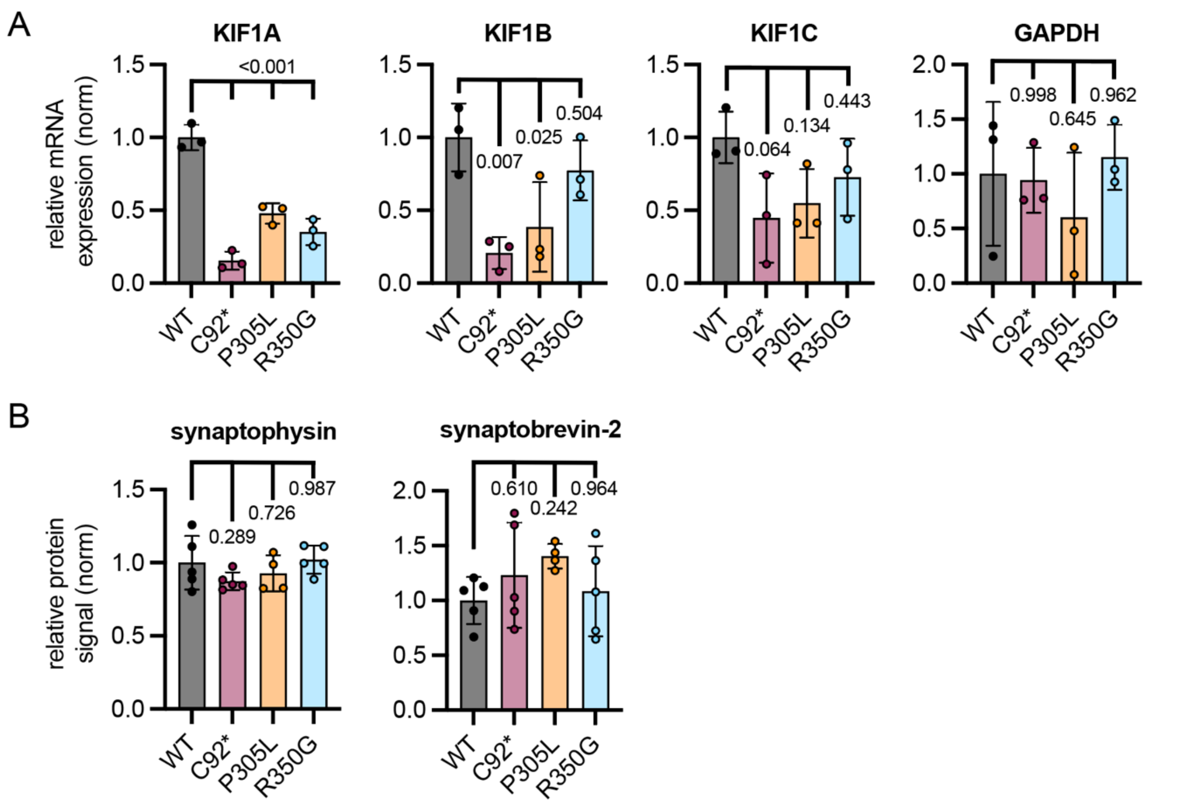

Figure S1

(A) RT-qPCR analysis reveals decrease in *KIF1A* mRNA level across *KIF1A* variant conditions. KIF1A null (p.C92*) also leads to decreased mRNA level of additional kinesin-3 motor family members, *KIF1B* and *KIF1C*. Housekeeping gene GAPDH was used as a reference control and wild-type condition was used to calculate relative fold change in kinesin-3 genes of interest.

(B) Quantification of relative protein levels of synaptophysin (SYP) and synaptobrevin-2 (SYB2) in DIV21 iNeuron lysate, corresponding to example western blot displayed in Figure 3A (mean ± standard deviation; n = 4 independent experiments; reported *p*-values are from ANOVA with multiple comparisons).

bioRxiv preprint doi: https://doi.org/10.64898/2026.01.14.699478; this version posted January 14, 2026. The copyright holder for this preprint (which was not certified by peer review) is the author/funder, who has granted bioRxiv a license to display the preprint in perpetuity. It is made available under aCC-BY 4.0 International license.

Supplemental Figure 2

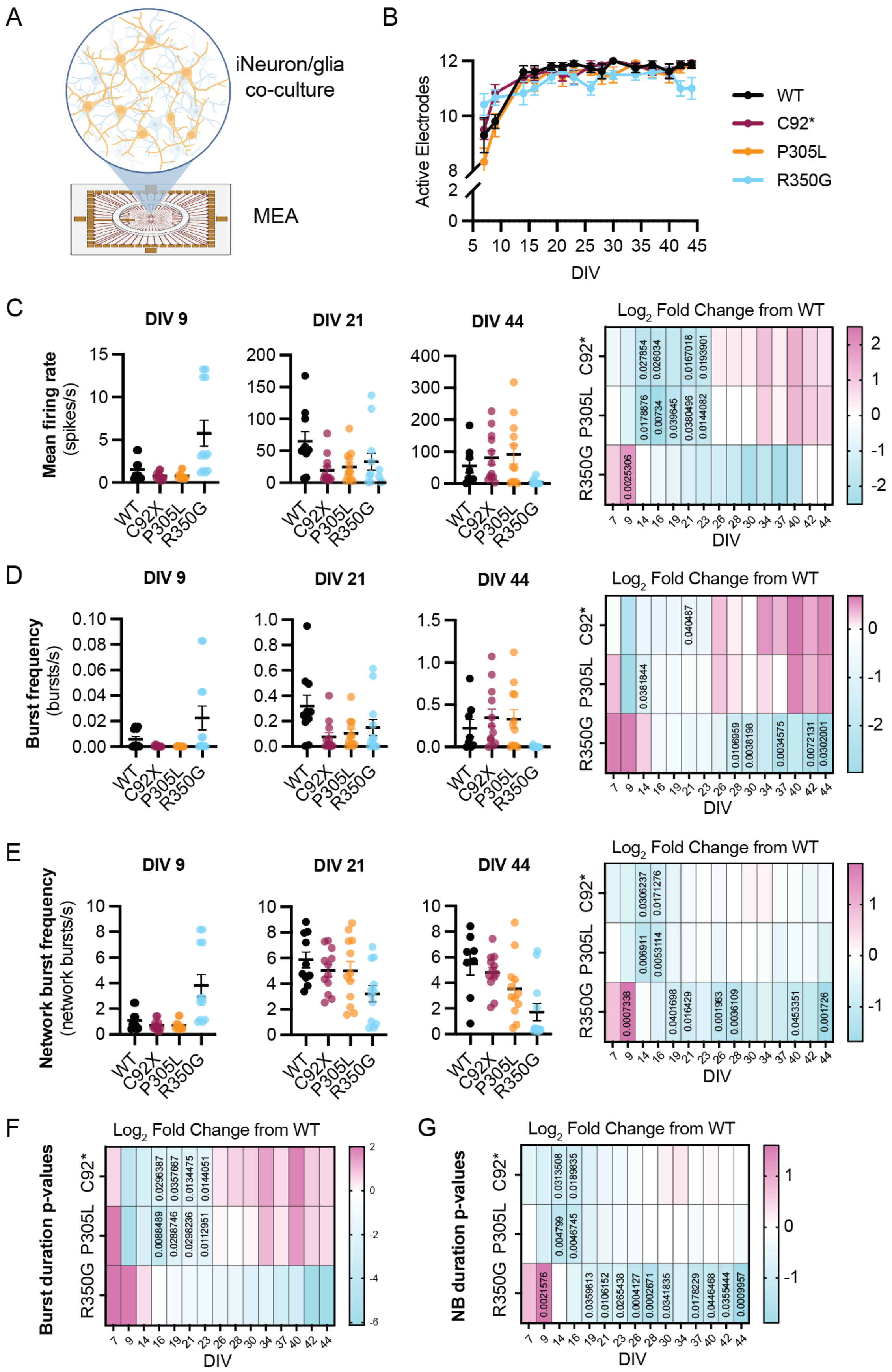

bioRxiv preprint doi: https://doi.org/10.64898/2026.01.14.699478; this version posted January 14, 2026. The copyright holder for this preprint (which was not certified by peer review) is the author/funder, who has granted bioRxiv a license to display the preprint in perpetuity. It is made available under aCC-BY 4.0 International license.

Figure S2

(A) Schematic of iNeuron/glia co-culture experimental set-up for spontaneous MEA recording.

(B) Longitudinal tracking of active electrodes for wild-type KIF1A iNeurons and p.C92*, p.P305L, p.R350G mutant iNeurons. Hyperactive p.R350G iNeurons exhibit an increase in active electrodes compared to wild type at early stages of culture.

(C-E) Mean firing rate (C), burst frequency (D) and network burst frequency (E) for KIF1A wild-type and mutant cultures at DIV 9, 21, and 44. Each dot represents an individual electrode, and error bars represent standard deviation. P-value heat map plots are provided to the right of individual time point plots.

(F, G) P-value heat map plots for burst duration (F) and network burst (NB) duration (G).

Heat map plots of p-values provide insight into significance across all recorded time points, with p-values < 0.05 provided in the corresponding box. Blue-shaded boxes reveal a decrease compared to wild type, and pink-shaded boxes reveal an increase compared to wild type.
